# **RETRACTION:** Treatment with β-elemene combined with paclitaxel inhibits growth, migration, and invasion and induces apoptosis of ovarian cancer cells by activation of STAT-NF-κB pathway: Fu Xiaomeng, Lv Lei, An Jinghong, Jiang Juan, Yue Qi, and Yuan Dandan Braz J Med Biol Res (2020) 53(6): e8885. http://dx.doi.org/10.1590/1414-431X20208885

**DOI:** 10.1590/1414-431X2025e8885retraction

**Published:** 2025-06-16

The Editors received the following communication from claire.francis1492@gmail.com: “More data in *Braz J Med Biol Res.* 2020;53(6):e8885. doi: 10.1590/1414-431X20208885. Epub 2020 May 8 appears very similar to data in *Oncotarget*. 2015 Sep 29;6(29):28296-311. doi: 10.18632/oncotarget.5064, although described differently”.

The corresponding authors, Dr. Yuan Dandan and Dr. Yue Qi, were contacted regarding this matter. They stated the following: “After thorough verification by the first author and the corresponding author, we identified the improper use of images in this manuscript, which resulted in a substantial similarity with the article published in *Oncotarget* (2015;Sep 29;6(29):28296-311). After consulting with all co-authors, we unanimously request the retraction of this article”.

In order to prevent further damage to the scientific community, the Editorial Team of the Brazilian Journal of Medical and Biological Research informs the formal retraction of this article.


Editorial Team - Brazilian Journal of Medical and Biological Research


